# Visual outcomes with a non-diffractive enhanced depth-of-focus IOL in patients with age-related macular degeneration

**DOI:** 10.3389/fmed.2025.1505401

**Published:** 2025-06-13

**Authors:** Juan Carlos Elvira, Patricia Devesa, Belén Elvira-Giner, Pedro Tañá-Sanz, Paz Orts-Vila, Pedro Tañá-Rivero

**Affiliations:** Oftalvist, Alicante, Spain

**Keywords:** age-related macular degeneration, enhanced depth-of-focus, cataracts, intraocular lens, patient satisfaction

## Abstract

**Purpose:**

To evaluate visual function in eyes with age-related macular degeneration (AMD) implanted with a non-diffractive enhanced depth-of-focus (EDOF) intraocular lens (IOL) after cataract surgery.

**Design:**

Prospective, observational, non-randomized clinical study.

**Methods:**

Twenty-two eyes from 22 patients diagnosed with AMD and cataracts were submitted to standard cataract surgery with a non-diffractive EDOF IOL implantation (AcrySof IQ Vivity). We measured monocular uncorrected and best-corrected-distance visual acuity (UDVA and CDVA), uncorrected- and distance-corrected-intermediate visual acuity (UIVA and DCIVA), uncorrected- and distance-corrected-near visual acuity (UNVA and DCNVA), manifest refractive spherical equivalent (MRSE) and cylinder, monocular defocus curve and patient-reported outcome questionnaires (Catquest-9SF and NEI VFQ-25). Follow-up visits were carried out at 1, 3 and 6 months post-surgery.

**Results:**

At 6 months post-surgery all eyes were within ± 0.50 D with a mean MRSE of −0.19 ± 0.20 D, 95.45% had a refractive cylinder of ≤ 0.50 D with a mean cylinder of −0.24 ± 0.27 D. The mean values of postoperative monocular CDVA, DCIVA, and DCNVA were 0.02 ± 0.08, 0.16 ± 0.11, and 0.26 ± 0.15 logMAR, respectively. The defocus curve showed good visual acuity at distance and intermediate with a depth-of-focus of about 1.60 D. A total of 81.82% of patients did not report any difficulty with their vision in their everyday-life and 86.36% reported being quite satisfied to very satisfied with their current vision. The NEI VFQ-25 showed that all values improved significantly (*p* < 0.05) after the surgery in the different parameters analyzed except for ocular pain (*p* = 0.390) and color vision (*p* = 0.333).

**Conclusion:**

The use of a non-diffractive EDOF IOL in AMD eyes with cataracts is a safe and effective surgical approach for visually correcting aphakia, providing good visual acuity at far and intermediate distances. Our outcomes support the use of non-diffractive EDOF IOLs in patients with AMD diagnosed with cataracts aiming to obtain spectacle-independence at far and intermediate distances.

## Introduction

Cataract surgery has been reported to effectively improve visual function in patients with age-related macular degeneration (AMD) (1–6). This surgery with IOL implantation is an appropriate solution in AMD patients with clinically significant cataracts. The severity of the AMD, and whether it is exudative or non-exudative, can lead to vision issues that impact intraocular lens (IOL) selection (7). However, the use of specific multifocal IOLs is often not considered for patients with certain retinal disorders, such as AMD, or at risk of developing these. These IOLs, using two or three focal points may reduce contrast sensitivity in healthy patients in some circumstances (8) and it has been argued that this reduction may be significant in eyes with pre-existing contrast sensitivity impairment, such as those with concurrent diseases (9). However, two studies have assessed the visual outcomes of multifocal IOLs in patients with AMD and concluded that a significant proportion of this type of patient benefits from the IOL’s multifocality (10); there is also no evidence to suggest that patients with AMD should be advised against using a multifocal IOL (11). Additionally, a recent review of multifocal IOLs and retinal diseases concluded that there is no evidence suggesting that patients with certain retinal diseases should be advised against multifocal IOLs (12). Those authors also pointed out the reduction in contrast sensitivity that should be considered to contraindicate the use of multifocal IOLs.

Enhanced depth-of-focus (EDOF) IOLs are lenses designed to elongate a single-focal-point to increase the area of focus and improve the quality of vision at different distances. Based on this technology, these lenses aim to reduce altered contrast sensitivity compared to traditional multifocal IOLs. However, there is some controversy about the possible difference between these two types of IOLs in terms of contrast sensitivity, since some studies consider that patients implanted with an EDOF have better contrast sensitivity values than those receiving trifocal IOLs (13), both either under photopic and scotopic conditions (14), while others have found comparable outcomes and no particular advantage of EDOFs over trifocal lenses in terms of contrast sensitivity (15–17). We therefore consider that the use of either an EDOF or trifocal IOL should be based on the surgeon’s judgment, taking into account the patient’s eye characteristics. We believe that a non-diffractive smooth surface is expected to obtain good visual outcomes without affecting contrast sensitivity in eyes with AMD and can allow good retinal fundus visualization that may be needed in these patients. It has been reported that a final corrected distance visual acuity (CDVA) of ≤ 0.3 logMAR is significantly associated with patient satisfaction in patients with neovascular AMD after cataract surgery (6). Providing good CDVA and, where possible, good vision at intermediate distances may be beneficial for daily visual tasks in AMD patients diagnosed with cataracts. A recent retrospective study using EDOF IOLs in patients with early AMD has shown that this type of IOL provides improved near vision proportional to far vision in these patients (18).

The aim of this clinical prospective study was to provide more clinical evidence on the use of the AcrySof IQ Vivity EDOF IOL in a series of eyes diagnosed with AMD and implanted with this model, through measuring visual acuity at different distances and assessing visual function using two patient-reported outcome questionnaires.

## Materials and methods

This study was done in a single center, being observational and prospective. It followed the Declaration of Helsinki, with all patients with the signed informed consent before. The Ethics Committee of the Hospital Clínico San Carlos in Madrid (Spain) and the Valencian regional committee on postmarketing studies CAEPRO in Valencia (Spain) approved the study. In addition, it was registered in the German Clinical Trials Register with the following number: DRKS00030673.

### Intraocular lens and surgery

All eyes were implanted with the AcrySof IQ Vivity EDOF IOL (Alcon Labs, Fort Worth, TX, United States). This model is a non-diffractive lens with ultraviolet and blue light filtering made of hydrophobic acrylate/methacrylate copolymer material (*n* = 1.55). The IOL has a biconvex wavefront-shaping optic for the spherical model and biconvex toric wavefront-shaping optic for the toric model. The optic diameter is 6.0 mm and the overall diameter is 13.0 mm. It presents a Stableforce modified-L haptics (haptic angle of 0 degrees). The spherical power of the lens is from + 10.00 to + 30.00 D and for toric lenses with powers of 1.00, 1.50, 2.25, 3.00, and 3.75 D. Standard phacoemulsification cataract surgery was performed through a 2.2 mm, clear, temporal corneal incision using a topical anesthetic and the Centurion^<reg>(</reg>^ vision system (Alcon Labs, Fort Worth, TX, United States) with a 5 mm diameter capsulorhexis.

### Patients and assessment

Patients underwent a full eye analysis, including preoperative CDVA, refraction, and anterior and posterior segment examination. The inclusion criteria were: age-related cataract surgery patients, candidates for AcrySof IQ Vivity with IOL power calculation ranging from + 10 to + 30 D, targeted to plano, patients, based on a fundus examination, macular optical coherence tomography (OCT) or autofluorescence, presenting mild pathology where a trifocal lens is not recommended for one or both eyes, drupes (drupelets or small drusen < 63 μm) in one or both eyes, early AMD with medium drusen of 63–125 μm without AMD-related pigment changes and pigment epithelium alterations without a geographic component, mild alteration observed in a macular OCT study, with partial loss of the ellipsoid line. The exclusion criteria were: advanced or intermediate AMD, other ocular co-morbidities or disease, and previous ocular surgeries.

The IOLMaster 700 biometer (Carl Zeiss Meditec AG, Jena, Germany) was used and the IOL power calculation was carried out using the Barrett Universal II formula, being emmetropia the target refraction. All patients were bilaterally implanted with the AcrySof IQ Vivity IOL (non-toric or toric model, as required) but only one eye per patient was considered for the analysis. If both eyes presented AMD, and were therefore eligible according to the inclusion and exclusion criteria, the eye included in the analysis was choose at random.

Three follow-up visits post-surgery were carried out (1, 3 and 6 months), being analyzed for the last post-operative visit. During these visits, we measured monocular logMAR uncorrected-distance visual acuity (UDVA), CDVA, uncorrected- and distance-corrected-intermediate visual acuity (UIVA and DCIVA, at 66 cm), and uncorrected- and distance-corrected-near visual acuity (UNVA and DCNVA, at 40 cm). subjective refraction, detailed by sphere, cylinder, and the manifest refraction spherical equivalent (MRSE), was recorded at all the postoperative visits, and double-angle tool (19) was used for vector analysis. At 6 months, we also recorded the monocular defocus curve (from + 1.00 to −3.00 D, in 0.50 D increments), to study the useful range of vision. Patients were also asked to complete two patient-reported-outcome questionnaires before surgery and at 6 months post-surgery: the Catquest-9SF and the 25-item National-Eye-Institute-Functional-Questionnaire (NEI VFQ-25), plus the additional questions in Appendix I. The first determines patient satisfaction and difficulties in daily life when carrying out certain activities using nine questions with four response options ranging from 4 (very great difficulty-very dissatisfied) to 1 for (no difficulty-very satisfied), and an additional option (cannot decide), which is treated as missing data. Its usefulness in cataract surgery patients has previously been reported (20–22). The NEI VFQ-25 measures vision-health-related-quality-of-life (23); it has been validated in different languages (24–26) and used in patients implanted with EDOF IOLs (27–29). This test generates different vision-targeted sub-scales. To obtain the score for the NEI VFQ-25, the instructions for the test were followed, converting each item to a 0–100 scale so that the lowest and highest possible scores were set at 0 and 100 points, respectively (the scores representing the achieved % of the total possible score, with 100% being the best possible score and 0% the worst). Also, surgical complications or adverse events were recorded.

### Sample size calculation and analysis

Based on a sample size of 22 eyes, a 95% confidence interval, and a standard deviation (SD) of 0.12 logMAR (30) for distance-visual-acuity, the precision for the primary outcome estimate is 0.07 logMAR. This is considered appropriate for the objective of this study. Mean, SD, and minimum and maximum values were considered for the descriptive analysis of the continuous variables and categorical variables were described as %. The Student’s *t*-test due to the normal distribution was used to compare the outcomes before and after the surgery according to the results of the NEI VFQ-25 questionnaire. The significance level was considered *p* < 0.05.

## Results

### Patients

We examined 22 eyes from 22 patients (14 males) diagnosed with AMD and cataracts. Table 1 shows the demographic and preoperative characteristics of the patients (73.9 years). The mean preoperative CDVA was 0.16 ± 0.14 logMAR. Eight eyes were implanted with the non-toric IOL model and 14 with the toric model (mean cylindrical IOL power 1.70 ± 0.79 D). No complications or adverse events were found either during the surgery or up to the final follow-up visit of the study.

**TABLE 1:** Demographics and preoperative measurements of participants shown as means, standard deviations (SD), and ranges.

	Mean value ± SD (range)
Eyes (*n*)	22
Sphere (D)	0.41 ± 2.72 (−6.25 to 5.25)
Refractive cylinder (D)	−1.10 ± 1.02 (−4.50 to 0.00)
Spherical equivalent (D)	−0.14 ± 2.60 (−6.80 to 4.88)
CDVA (logMAR)	0.16 ± 0.14 (0.00 to 0.50)
K1 (D)	43.79 ± 1.27 (41.34 to 46.14)
K2 (D)	44.81 ± 1.33 (41.98 to 47.35)
Axial length (mm)	23.33 ± 1.01 (21.36 to 26.27)
ACD (mm)	3.12 ± 0.29 (2.63 to 3.59)
IOL spherical power (D)	21.50 ± 2.89 (13.00 to 28.00)
IOL cylindrical power (D)	1.70 ± 0.79 (1.00 to 3.75)

CDVA, corrected distance visual acuity; K, keratometry; ACD, anterior chamber depth; IOL, intraocular lens.

### Refraction

**FIGURE 1:**
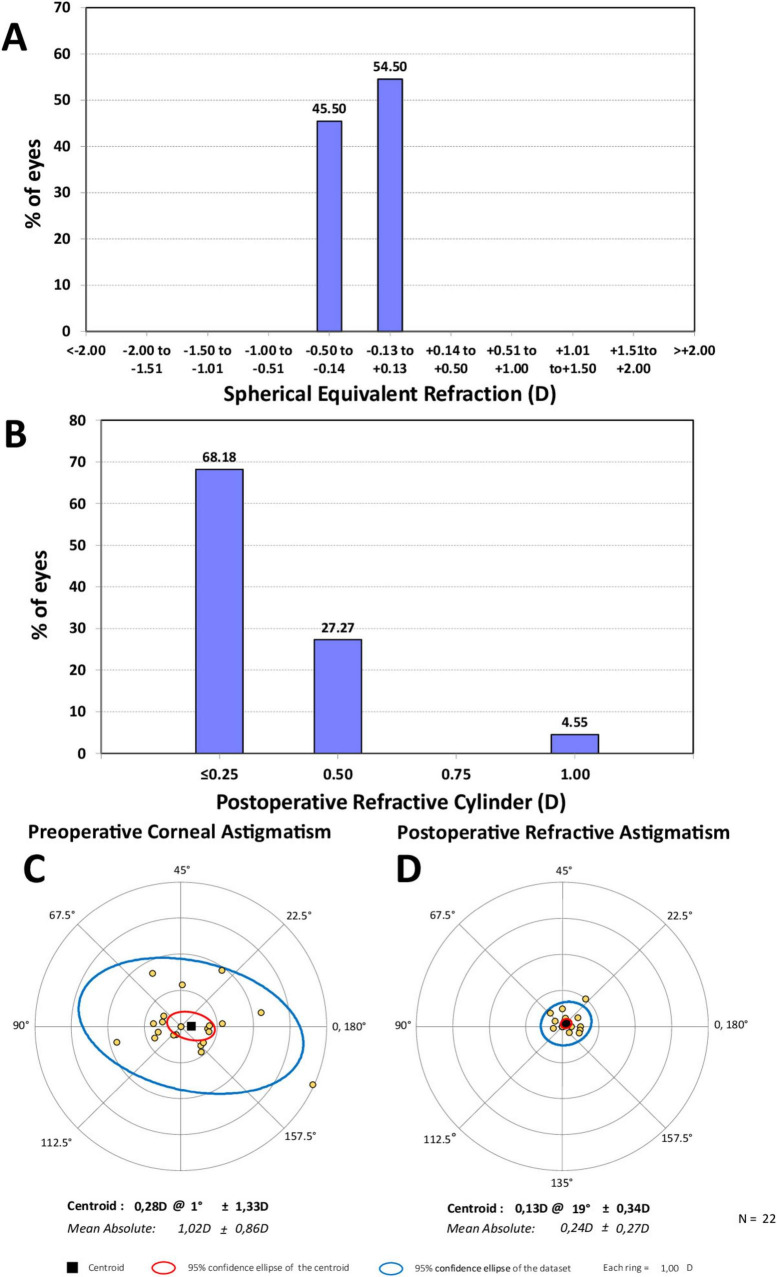
Distribution of spherical equivalent refraction **(A)** and refractive cylinder **(B)** 6 months post-surgery, and double-angle plots for preoperative corneal astigmatism **(C)** and postoperative refractive astigmatism **(D)** 6 months post-surgery applying the double-angle tool. Centroids, mean absolute values with standard deviations, and 95% confidence ellipses of the centroid and dataset are also shown.

Figure 1A shows the distribution of MRSE post-surgery indicating that 54.50% of eyes (*n* = 12) were within ± 0.13 D and 45.50% (*n* = 10) were in the range −0.14 to −0.50 D. All the implanted eyes were within ± 0.50 D. The mean MRSE was −0.19 ± 0.20 D, ranging from −0.50 to 0.00 D. The analysis of the refractive cylinder in Figure 1B revealed that 68.18% (*n* = 15) of eyes were within ≤ 0.25 D and 95.45% (*n* = 21) were within ≤ 0.50 D, the mean refractive cylinder being −0.24 ± 0.27 D, ranging from 0 to −1.00 D. Double-angle plots of are shown in Figure 1C for the preoperative corneal astigmatism and in in Figure 1D for the postoperative refractive astigmatism. The mean absolute preoperative corneal astigmatism was 1.02 ± 0.86 D and the mean absolute postoperative refractive astigmatism was 0.24 ± 0.27 D.

### Visual acuity at different distances

**FIGURE 2:**
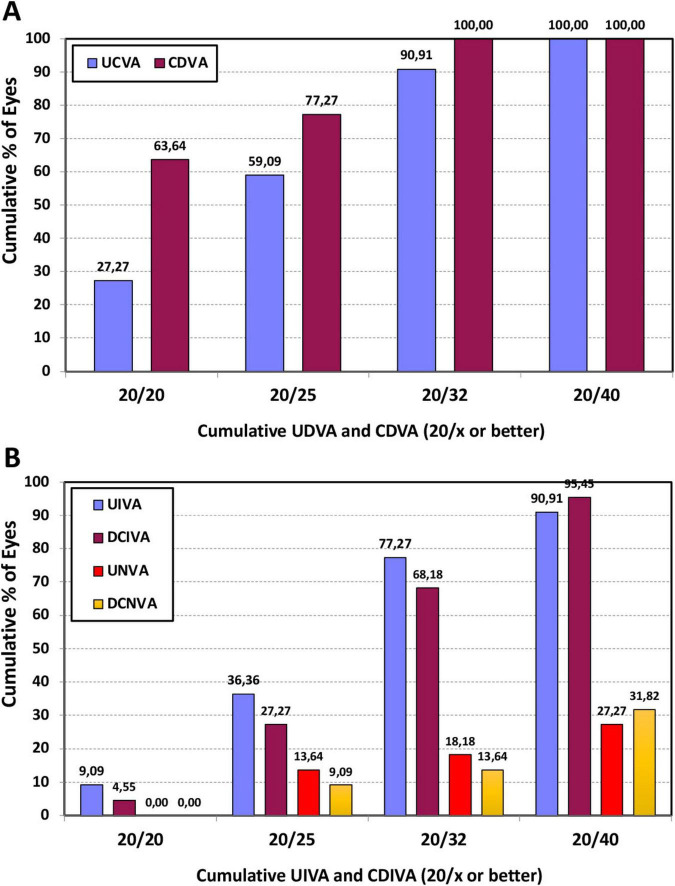
Cumulative percentage of eyes at 6 months post-surgery with different degrees of uncorrected and best-corrected distance visual acuity (UDVA and CDVA) **(A)**, and uncorrected and distance-corrected intermediate visual acuity at 66 cm (UIVA and DCIVA) and uncorrected and distance-corrected near visual acuity at 40 cm (UNVA and DCNVA) **(B)**.

**FIGURE 3:**
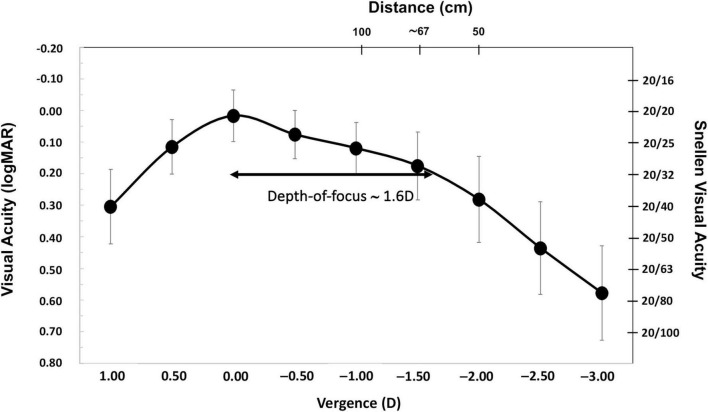
Mean monocular logMAR visual acuity with best correction for distance based on the vergence chart for AcrySof IQ Vivity) intraocular lens (IOL) at 6 months post-surgery. The error bars show the standard deviation. The right y-axis shows the Snellen visual acuity in feet and the top x-axis is the distance (cm). Depth-of-focus was defined as the range of lens powers that achieved a mean acuity of 20/32 or better (from 0 D of vergence).

With regard to the visual acuity outcomes, Figure 2 provides the cumulative percentage of eyes that achieved given monocular UDVA and CDVA values (A), and UIVA, DCIVA, UNVA, and DCNVA scores (B) at 6 months post-surgery. The CDVA was ≥ 20/25 in 77.27% (*n* = 17) of eyes and ≥ 20/32 in 100% (*n* = 22). The DCIVA was ≥ 20/25 in 27.27% (*n* = 6) of eyes and ≥ 20/32 in 68.18% (*n* = 15), while the DCNVA was ≥ 20/32 in 13.64% (*n* = 3) and ≥ 20/40 in 31.82% (*n* = 7) of eyes. The average values for the postoperative monocular UDVA, UIVA, and UNVA were 0.08 ± 0.09, 0.15 ± 0.12, and 0.33 ± 0.14 logMAR, respectively. For corrected distance, CDVA, DCIVA, and DCNVA, these values were 0.02 ± 0.08, 0.16 ± 0.11, and 0.26 ± 0.15 logMAR, respectively. Figure 3 depicts the mean monocular defocus curve, with a peak for far vision (0 D), followed by a steady reduction with negative vergences corresponding to intermediate and near vision. The depth-of-focus was defined as the lens power range that achieved a mean acuity of ≥ 20/32 from 0 D, which for our results it was about 1.60 D.

### Patient-reported outcomes questionnaires

**FIGURE 4:**
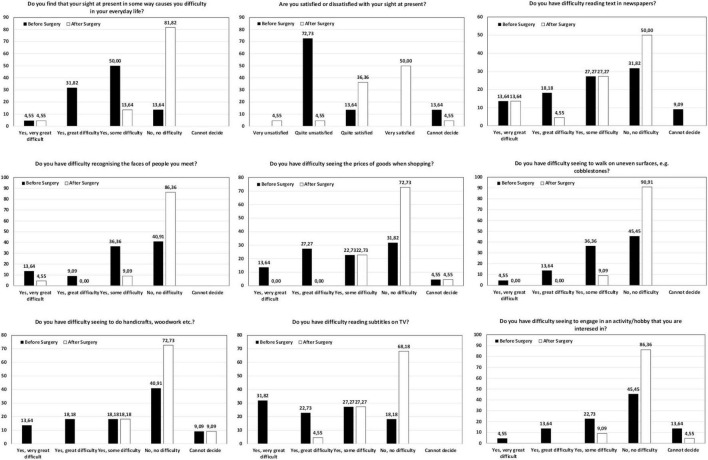
Distribution of the answers (percentage) for the different questions in the Catquest-9SF questionnaire before and after the surgery.

**FIGURE 5:**
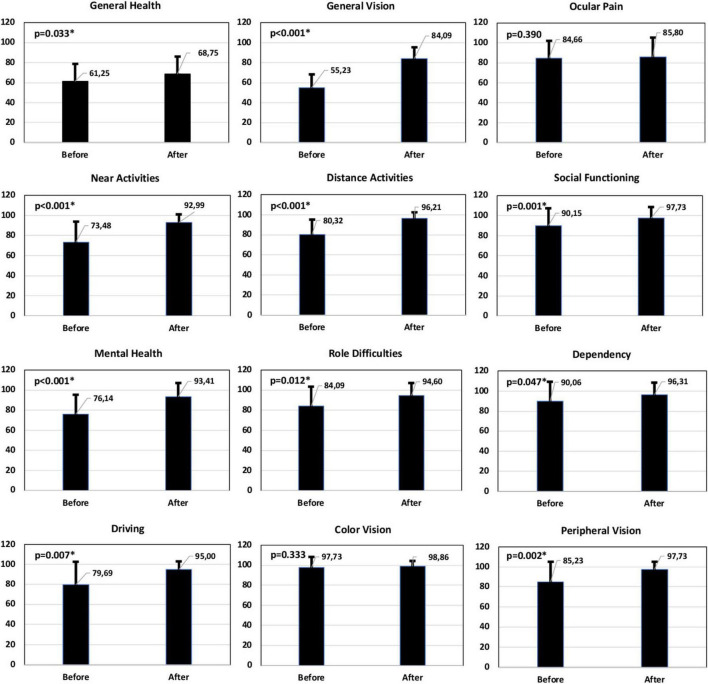
Mean and standard deviation NEI VFQ-25 score (percentage) for different vision-targeted sub-scales and a single general health rating question before and after the surgery. Note that the scores represent the achieved percentage of the total possible score, with 100% being the best and 0% the worst possible score. The Student’s *t*-test was conducted to evaluate the significance of the differences between before and after the surgery. The asterisk * indicates a statistically significant difference (*p* < 0.05).

Patients were asked to answer the Catquest-9SF and NEI VFQ-25 questionnaires prior to their surgery as well as at 6 months post-surgery. Figure 4 shows the distribution of the answers in percentages for the different questions on the Catquest-9SF questionnaire pre- and post-operatively, summarizing the patient-reported limitations in certain daily activities and their satisfaction with their current vision. A total of 81.82% of patients reported having no difficulties in their everyday life. A total of 86.36% of patients reported being quite satisfied to very satisfied. For various specific tasks, between 50% and 90.91% of patients reported no difficulty performing them, with reading text in newspapers presenting the lowest value. Figure 5 shows the NEI VFQ-25 scores (mean and SD) before and after surgery for the different vision-targeted questions and a health rating question. Note that all values improved significantly (*p* < 0.05) after the surgery for the different parameters analyzed except for ocular pain (*p* = 0.390) and color vision (*p* = 0.333), where no differences were reported.

## Discussion

We demonstrate the effectiveness of cataract surgery with a non-diffractive EDOF IOL implantation in AMD patients. The visual acuity outcomes reveal that patients show mean CDVA, DCIVA, and DCNVA values of 0.02 ± 0.08, 0.16 ± 0.11, and 0.26 ± 0.15 logMAR, respectively. The design of the lens offers an extended range of vision, particularly for intermediate vision graphically described in Figure 3 (note that the lens offers a depth-of-focus of about 1.6 D). Our results reveal excellent refractive outcomes, in both MRSE and astigmatism correction (see Figures 1A, B), with 100% of eyes being within ± 0.50 D of MRSE and a mean postoperative MRSE of −0.19 ± 0.20 D and 95.45% of eyes with a refractive cylinder of ≤ 0.50 D and a mean postoperative value of −0.24 ± 0.27 D. The reduced postoperative refractive astigmatism, shown in Figure 1D, should also be noted. Our results showed similar refractive and visual acuity values to healthy eyes implanted with this IOL model (31–33). For example, the multicounty study of Bala et al. (31) analyzed 156 patients implanted with this lens (non-toric) at 6 months post-surgery and found that close to 85% of patients achieved a mean MRSE of ≤ 0.50 D (84.7%, mean of −0.15 ± 0.32 D) and mean monocular values of −0.008 ± 0.007, 0.161 ± 0.013, and 0.414 ± 0.013 logMAR, for CDVA, DCIVA, and DCNVA, respectively. Similarly, McCabe et al. (32), in 107 patients also implanted with the non-toric IOL, also reported that at 6 months 91.6% of eyes achieved a MRSE within ± 0.50 D (mean 0.049 ± 0.345 D) with a mean monocular value of 0.016 ± 0.009, 0.148 ± 0.012, and 0.359 logMAR for CDVA, DCIVA, and DCNVA, respectively. Specifically, the toric model in eyes with low corneal astigmatism, Pastor-Pascual et al. (33) looked at 47 eyes implanted with the AcrySof IQ Vivity Toric T2 at 3 months and found that 100% of eyes had a MRSE within ± 0.50 D (mean −0.10 ± 0.17 D), and mean values of −0.02 ± 0.08, 0.14 ± 0.09, and 0.23 ± 0.12 logMAR for CDVA, DCIVA, and DCNVA, respectively. The defocus curves in these studies showed similar outcomes, for example, Bala et al. (31) determined, in binocular conditions, that patients achieved ≤ 0.0 logMAR from + 0.50 to −0.50 D, < 0.1 logMAR down to −1.50 D, and < 0.2 logMAR down to −2.00 D; McCabe et al. (32) found an increase of 0.54 D at 0.2 logMAR under monocular conditions compared to the monofocal AcrySof IQ IOL; and Pastor-Pascual et al. (33) reported a monocular depth-of-focus of about 1.75 D in their cohort.

Our patient-reported questionnaires revealed good outcomes in terms of satisfaction (86.36% quite satisfied-very satisfied) and difficulties when performing various visual tasks, as per Catquest-9SF (see Figure 4). This correlates with the outcomes of the NEI VFQ-25 questionnaire with post-surgery improvement being reported for the main parameters analyzed (see Figure 5). It is interesting to note the improvement in near (73.48 versus 92.99, *p* < 0.001) and distance activities (80.32 versus 96.21, *p* < 0.001) and driving (79.69 versus 95, *p* = 0.007) after the surgery. Rementería-Capelo et al. (34) analyzed patient satisfaction in 25 patients with ocular pathologies after AcrySof IQ Vivity EDOF IOL implantation using the Catquest-9SF questionnaire (six patients with glaucoma; four with cornea guttata; three patients with dry AMD; two each with amblyopia, ocular hypertension, and corneal leucoma; and one with epiretinal membrane, macular telangiectasia, lagophthalmos, homonymous hemianopia, previous LASIK surgery and daltonism). In a comparison with a healthy control group of patients implanted with the same lens, they found the coexisting pathology group showed a higher level of satisfaction than patients in the control group (*p* = 0.016), and patients in the control group reported higher difficulties reading newspapers (*p* = 0.030). The authors indicated that there were no other significant differences between groups and patients indicated they would undergo the surgery again using the same IOL. They also indicated that their main limitation in the study was the wide range of ocular pathologies included and the low number of each pathology. Labiris et al. (35) analyzed 30 patients implanted bilaterally with the toric and non-toric Vivity IOL and analyzed the outcomes at 6 months post-surgery, using the NEI-VFQ-25 questionnaire. They found mean values for total, near and distance activities of 87.56 ± 8.89, 85.77 ± 9.72, and 88.73 ± 10.34, respectively (see Figure 5 for a comparison with our results). These authors compared this group of patients with two other groups, with bilateral PanOptix IOL and mix-and-match, reporting significant better outcomes for these two groups compared to the patients with bilateral Vivity IOLs.

Few studies have analyzed the use of presbyopia-correcting IOLs in patients with AMD. Two studies analyzed the implantation of multifocal IOLs in this type of patient; we know that a direct comparison with our outcomes is not possible due to the different IOL design, but we do consider it interesting to discuss the results. The first study reported the outcomes of 36 AMD eyes implanted with Array multifocal refractive IOLs and compared these with a control group that received monofocal IOLs (10). The authors concluded that the Array IOL provides distance vision comparable to those of the monofocal IOL and found a significant percentage of these patients benefited from the IOL’s multifocality (10). In relation to complementary procedures, they indicated that retinal visualization was not impaired, and fluorescein angiography and laser photocoagulation could be performed without difficulty when required in eyes with multifocal IOLs (10). Note that this is not expected with the Vivity IOL due to its design. In this sense, Al-Amri et al. (36) have evaluated the clinical retinal image quality of different IOLs and found that the Vivity IOL showed comparable outcomes to the monofocal AcrySof SA60AT (*P* > 0.05). These authors indicated that the Vivity IOL performs similarly to monofocal IOLs in relation to the *in vivo* clinical retinal optical image quality, without any measurable compromise from the addition of the wavefront-shaping technology of this lens (36). In the other study, Gayton et al. (11) implanted the bifocal diffractive AcrySof ReSTOR IOL targeting −2.0 D in eyes with AMD and a CDVA of 20/50 or worse to provide an uncorrected near of + 5.2 D. This was a specific multifocal-magnification strategy. They examined 20 eyes 6 months after the surgery and found a CDVA improvement in 14 eyes (70%) and improved CNVA in 17 eyes (85%). These authors administered the VFQ-25 questionnaire and found that all patients (*n* = 13) reported a significant improvement in visual-related items but not general health. Specifically, the score changes from preoperative levels to 6 months post-surgery were the following: general health (−8 ± 16), general vision (24 ± 14), ocular pain (5 ± 18), difficulty with near-vision activities (15 ± 31), difficulty with distance-vision activities (14 ± 24), limitations in social-functioning (13 ± 25), mental health (23 ± 28), role limitation (19 ± 29), dependency (18 ± 31), driving difficulties (11 ± 32), limitations with color vision (4 ± 29), and limitations with peripheral-vision (16 ± 16). These authors concluded that their preliminary results suggest that this procedure holds promise for the visual rehabilitation of AMD eyes with cataracts. Our results do not consider this type of strategy providing our patients good distance and intermediate visual acuity not using diffractive designs that may affect retinal visualization (see defocus curve plotted in Figure 3).

As we have mentioned, a retrospective-study using the Vivity IOL in patients with early AMD has been published (18). Thananjeyan et al. (18), in a 2 years pilot study assessed 51 eyes (28 patients) with seven early, 17 intermediate and 27 late-stage AMD based on the Beckman clinical-classification implanted with the AcrySof IQ Vivity IOL. Of eyes with late AMD, 17 had wet AMD. They reported a postoperative monocular CDVA and DCNVA at 50 cm of 0.20 ± 0.25 logMAR and N9 (range N5/N36), respectively. A total of 6/5–6/6 Snellen CDVA was found in 29.4% of eyes, and 6/7–6/12 Snellen CDVA in 52.9% of eyes; and 15.7%, 31.4% and 29.4% of eyes had near visual acuities of N6, N8, and N10, respectively. In addition, they measured quality of life using the VF-14 questionnaire and found that all patients reported improvement in daily-activities after the surgery, with 75% of patients reporting no symptoms of dysphotopsia in routine-clinical follow-up visits. The authors also indicated dysphotopsia was not reported to be a limiting factor, and 96% were satisfied with the degree of spectacle-independence and their quality of life post-IOL implantation (with primary spectacle use being for fine near vision tasks). In this cohort, all eyes with clinically classified early and intermediate AMD were able to achieve functional-near-visual-acuity, and eyes with clinically classified late AMD showed a larger spread of CDVA and DCNVA (18). The authors suggested that this could be due to greater variability in visual impairment with disease progression and/or secondary to anti-VEGF therapy in eyes with wet AMD. These authors also measured contrast sensitivity and found that patients achieving satisfactory vision, with Snellen levels of 6/5–6/12, had a contrast-sensitivity within the low normal range, and lower values were obtained in patients with more advanced stages of AMD who had a poorer CDVA. They concluded that the use of this IOL model in these patients allows a range of spectacle free vision and adds a range of satisfactory near and intermediate vision that would not be achieved with a monofocal IOL implantation. They indicated that this lens should be considered in clinical practice for patients with disease, thereby affording them the benefits of multifocality that patients without AMD achieve, while preserving contrast-sensitivity. We broadly agree with them and our outcomes support the use of this lens (18).

We should consider the following limitations of our study: relatively low number of participants, 6 months follow-up, and the lack of contrast sensitivity measurements and a control group to compare the outcomes obtained. However, we have discussed our findings in light of the outcomes reported in previous work in healthy eyes implanted with the same EDOF IOL, and the subsequent follow-up. We believe that despite of not considering a direct control group to compared directly the outcomes reported in our series, the comparison with previous literature on healthy eyes published is valid since the examination protocol and tests were similar or the same in some metrics. Then, can be directly compared in this sense. However, we consider that future studies should include normal healthy patients and eyes with different AMD severities, and, as it is a new procedure, longer follow-ups are required to support long-term safety levels.

In conclusion, the outcomes of our study suggest that cataract surgery with non-diffractive EDOF IOL implantation in AMD patients is satisfactory and efficient in terms of providing good visual acuity at far and intermediate distances. We, therefore, support the use of the AcrySof IQ Vivity in patients with AMD diagnosed with clinically significant cataracts in an aim to obtain spectacle independence at far and intermediate distances.

## Data Availability

The raw data supporting the conclusions of this article will be made available by the authors, without undue reservation.
